# Comparison of Temporalis Fascia and Full-Thickness Cartilage Palisades in Type-I Underlay Tympanoplasty for Large/Subtotal Perforations

**Published:** 2017-03

**Authors:** Pradeep Pradhan, Abhimanyu Anant, Vellore-Pattabhiram Venkatachalam

**Affiliations:** 1 *Department of Otorhinolaryngology, All India Institute of Medical Sciences, Bhubaneswar, Odisha, India.*; 2 *Department of Otorhinolaryngology,Safdarjung Hospital & Vardhmann Mahavir Medical College, Ansari Nagar, New Delhi, India.*

**Keywords:** Cartilage, Temporalis fascia, Tympanoplasty

## Abstract

**Introduction:**

To demonstrate surgical techniques and to compare the anatomical and functional outcomes between temporalis fascia and cartilage palisade grafting in type-I underlay tympanoplasty in patients with large/subtotal perforation.

**Materials and Methods:**

Temporalis fascia and cartilage palisade grafting were conducted in Group A and Group B, respectively, each containing 30 patients with large/subtotal perforations. Pure tone audiogram (PTA) and speech reception thresholds (SRT) were performed preoperatively and at each postoperative visit; i.e. at the end of Month 1,3,6, and 24. A 10-dB closure of air bone gap (ABG) and a 10-dB improvement in SRT were considered significant.

**Results:**

The graft uptake rates were 80% and 96.7% in Group A and Group B, respectively, at the end of 24 months. In total, 90% of Group A and 88% in Group B had significant improvement in hearing (ABG ≥10 dB). The mean improvement in SRT in the fascia and cartilage groups was 10 dB and 9 dB, respectively. Seventy-five percent of patients in Group A and 60% of patients in Group B had a significant gain in SRT.

**Conclusion:**

Although both temporalis fascia and cartilage palisades can effectively be used for tympanic membrane (TM) grafting in difficult perforations, the latter is considered to be the better autograft, not only because of superior graft uptake but also because it results in a comparable hearing outcome.

## Introduction

Although temporalis fascia graft is considered to be the gold standard for the repair of tympanic membrane (TM) defects in chronic otitis media (COM), it is challenging in the cases of subtotal/large perforations due to its poor graft uptake rate. This could be due to the minimal presence of residual tympanic membrane (TM) left for the lateral support of the graft. Further, the absence of a fibrous layer in the healed neotympanum makes it vulnerable for retraction with negative mesotympanic pressure, which may further lead to recurrence of the disease over a long period of time. To overcome this poor surgical outcome, autologous cartilage graft (conchal/tragal) has been used in previous decades by various researchers, showing encouraging postoperative results (1,2).

Being widely available, cartilage can be harvested in ample quantities from the same surgical site as the temporalis fascia graft, and later can be used in the form of island or cartilage palisades for the repair of a TM defect. Due to its tensile strength, cartilage can withstand the pressure changes in the mesotympanum; hence it is preferred over temporalis fascia for the reconstruction of TM defects, in which eustachian tube dysfunction is thought to be an important etiological factor. Further, it has been documented that cartilage is nourished by simple diffusion by incorporating it into the surrounding tissue (3), where it can survive in a relatively avascular condition, increasing its graft uptake rate in contrast with the temporalis fascia graft. Hence these are considered the ideal autografts, particularly in COM with large/subtotal perforations. Our aim is to compare the long-term anatomical and functional outcomes of type-I cartilage tympanoplasty using temporalis fascia graft and full thickness cartilage palisades in patients with COM with large/subtotal central perforations.

## Materials and Methods

This was a prospective study conducted in the Department of Otolaryngology at Safdarjung Hospital from July 2012 to June 2015. A total of 60 patients with COM with large/subtotal perforations were included in the study. All patients underwent type-I tympanoplasty. Temporalis fascia grafts were used in 30 patients (Group A) and full thickness cartilage palisades were used in the remaining 30 patients (Group B). Patients with ossicular chain discontinuity, conductive hearing loss due to acquired and congenital conditions other than tympanic membrane perforation, sensorineural deafness, and revision surgeries were excluded. Pure tone audiogram (PTA) and speech reception thresholds (SRT) were performed preoperatively in each patient.

*Surgical technique:*

Patients were operated on under local anesthesia using a post aural/endaural approach. Temporalis fascia/cartilage grafts (tragus/ concha) were harvested. Special care was taken to preserve the dome of the tragus while harvesting the tragal cartilage in order to avoid cosmetic deformity. Margins of the perforations were freshened. Along with the fibrous annulus, a circumferential tympanomeatal flap was elevated all around from the bony annulus, keeping it pedicled between 12 and 1 o’clock anterosuperiorly. Ossicular continuity was assessed. In patients in Group A, underlay grafting of the temporalis fascia was carried out by extending it anterosuperiorly over the lateral wall of the Eustachian tube and securing medially by placing gel foams in the Eustachian tube and in the middle ear. The tympanomeatal flap was then reposited back after ensuring that the flap and the graft were closely approximated to each other circumferentially.

**Fig 1:**
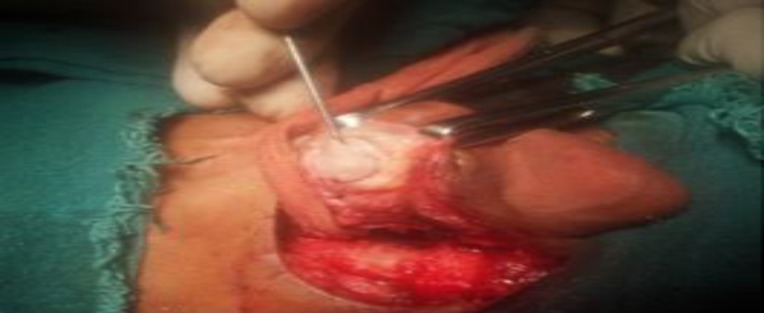
Conchal cartilage harvested through the postaural approach

**Fig 2:**
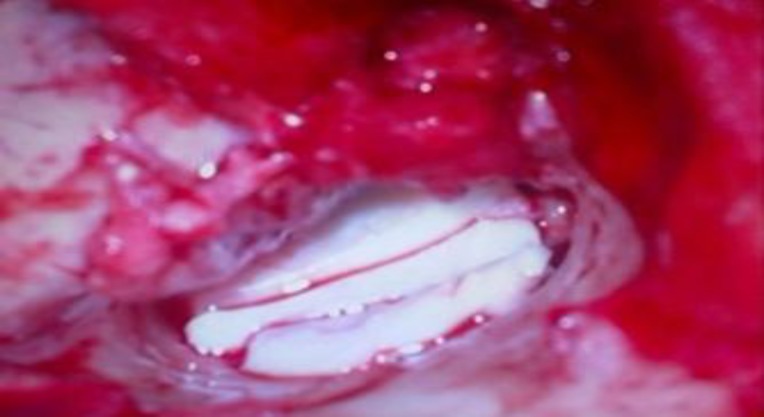
Full thickness cartilage palisades were placed parallel to the handle of the malleus

For patients in Group B, conchal/tragal cartilages were harvested through postaural and endaural approaches, respectively (Fig.1). The perichondrium on one side was removed from the cartilage and was divided into full thickness strips and trimmed approximately to the size of the TM. One parallel strip was placed anterior to the handle of malleus, securing anterosuperiorly against the lateral wall of the Eustachian tube. Another strip was placed posterior to the handle of malleus, and the third was placed between them (Fig. 2). 

Care was taken not to override the cartilage to the posterior meatal wall, and the cartilage assembly was made to fit snugly in the middle ear by placing a large piece of gel foam medially. The perichondrium layer was spread over the cartilage palisade laterally, and the tympanomeatal flap was then reposited back securing the margin of the flap over the perichondrium. The external auditory canal was packed with medicated gel foam. The postaural/endaural incision was closed with double-layer suturing and a dressing was placed. Patients were discharged after 24 hours of observation in the postoperative period.

The first postoperative visit was advised after 1 week for removal of the aural pack and sutures. Afterwards, patients were advised to visit the outpatient department at the end of Months 1, 3, 6, and 24 after surgery. Pure tone audiogram (PTA) and speech reception threshold (SRT) were carried out in each patient postoperatively during each follow-up visit. Air conduction and the bone conduction threshold were calculated at frequencies of 500, 1,000, 2,000, and 4,000 Hz. 

**Fig 3:**
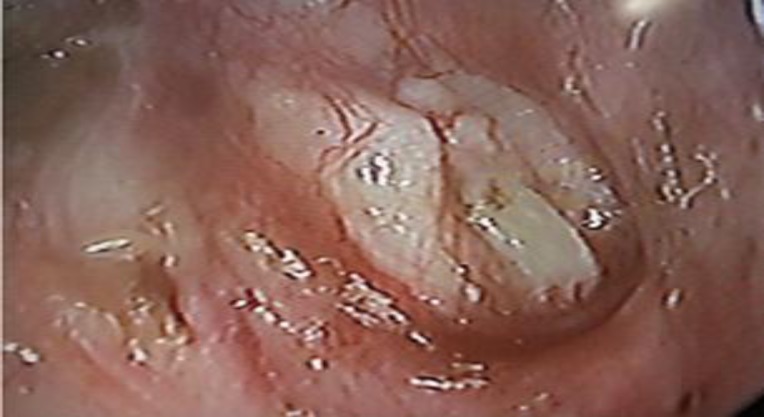
Otoendoscopic view showing a healed TM after cartilage palisade grafting

The preoperative audiogram was compared with the final postoperative audiogram, and ≥10-dB improvement in both air bone gap (ABG) in the air conduction threshold and SRT were considered significant. Otomicroscopic examination of the operated ears was carried out in each follow-up visit to assess the graft uptake (Fig.3) and complications that would have occurred in the follow-up period. Patient data were analyzed statistically using the Studentt-test. All procedures performed in studies involving human participants were in accordance with the ethical standards of the institutional and/or national research committee and with the 1964 Helsinki declaration and its later amendments or comparable ethical standards.

## Results

Of 60 patients, 38 (63.3%) were male and 22 (36.7%) were female. Type-I tympanoplasty using temporalis fascia graft and cartilage palisades were performed in Group A and Group B, respectively. Demographic data and results are described in Table 1.

**Table 1:** Description of preoperative and postoperative data in the study population

	**Temporalis fascia grafting**	**Cartilage palisade grafting**	**All patients**
Male	20	18	38
Female	10	12	22
Age (year)			
Mean	28	30	29
Range	17-32	19-40	17-40
Follow-up (month)			
Mean	24	24	24
Range	12-32	15-36	12-36
Surgical approach			
End aural	9	12	21
Post aural	21	18	39
Surgical outcomes.			
Graft uptake rate	80%	96.66%	83.33%
Preoperative PTA	30.23 ±7.77dB	29.06 ± 7.2 dB	29.40±8.29dB
Postoperative PTA	14.60 ± 4.6 dB	17.40 ±6.4 dB	15.71±5.71dB
SRT (speech reception threshold)			
Preoperative (dB)	25	29	27
Postoperative (dB)	15	20	17

The graft uptake rate in patients in the temporalis fascia group (Group A) was found to be 79%; i.e. of 30 patients, seven had residual perforation observed after 1 month of surgery. One of the perforations was found to be healed at the end of Month 6 with conservative treatment. At the end of Month 24, only 24 patients attended the otologic clinic and the remaining six patients were assessed over the telephone. It was found that eight patients had residual perforation at the end of Month 24, and the final graft uptake rate was 73.3%. Similarly, in Group B, 28 (93.3%) patients presented with complete closure of the perforation at the end of Month 1, with the failures resulting from the displacement of cartilage palisades. At the end of Month 6, one of the perforations was healed with conservative management. Only 22 patients were able to attend the otology clinic at the end of Month 24, and the remaining eight patients were followed by telephone. The final success rate was found to be 96.7%; i.e. one patient had residual perforation at the end of Month 24. The mean preoperative and postoperative air conduction hearing threshold in the temporalis fascia group (Group A) were found to be 30.23 ±7.77 dB and 14.60 ± 4.6 dB, respectively, with 15.63-dB closure of the ABG. Sixty percent of the patients achieved ≥15-dB closure of the ABG (18). In 30% of the patients (9), ABG closure was between 10 and 15 dB, while in 10% of patients (3), improvement in hearing was insignificant (ABG˂10dB). In the cartilage tympanoplasty patients (Group B), the mean preoperative and postoperative air conduction thresholds were found to be 29.06±7.2 dB and 17.40±6.4 dB, respectively, with 11.66-dB closure of the ABG. 48%(14) of the patients presented with ≥15 dB closure of ABG, while 40% showed closure of ABG between 10 and 15 dB and the remaining 12%(4) of patients showed insignificant improvement in hearing (ABG ˂10 dB).

ABG was found to be improved at each frequency level in both Group A and Group B (i.e., 500, 1,000, 2,000, and 4,000 Hz). The mean preoperative ABGs with corresponding frequencies were 25, 20, 35, and 40 dB in Group A compared with Group B where corresponding values were 3, 36, 25, and 23 dB. Similarly, mean postoperative ABGs were 14, 15, 10, and 18 dB in Group A and 22, 29, 10, and 07 dB in Group B, respectively (Table.2). The mean improvement in ABG was 11, 5, 15, and 22 dB in Group A and 10, 07, 15, and 18 dB in Group B. Further, the mean preoperative and postoperative SRT were 25 and 15 dB in Group A and 29 and 20 dB in Group B. The mean improvement in SRT in the fascia and cartilage groups were 10 dB and 9 dB, respectively. In total, 75%(22) of patients in Group A and 60% (18) of patients in Group B showed significant improvement in the SRT (≥10dB). No significant intraoperative complications were noted in the procedures in patients of either group, and no patient was found with lateralization of the graft or blunting of the anterior angle.

**Table 2:** Shows the closure of mean ABG in patients with temporalis fascia and cartilage palisades tympanoplasty at frequency 500 Hz, 1000 Hz, 2000 Hz and 4000 Hz

**ABG**	**500Hz**	**1000Hz**	**2000Hz**	**4000Hz**
Preoperative ABG in fascia group (dB)	25	20	35	40
Preoperative ABG in cartilage group (dB)	32	36	25	23
Postoperative ABG in fascia group (dB)	14	15	10	18
Closure of the ABG (dB)	11	05	25	22
Postoperative ABG in cartilage group (dB)	22	29	10	07
Closure of the ABG (dB)	10	07	15	16

## Discussion

**Fig 4:**
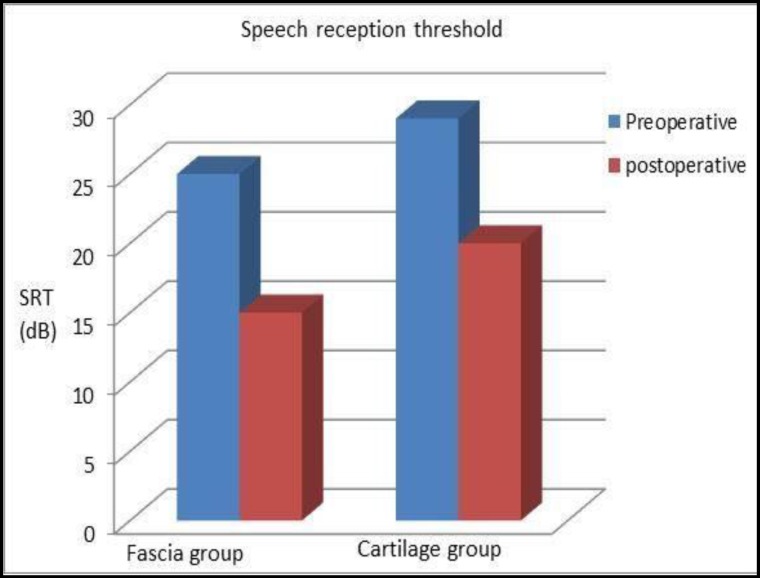
Improvement in SRT compared between the temporalis fascia graft and cartilage palisade tympanoplasty

Type-I tympanoplasty is one of the most common surgical procedures performed in any otologic clinic for the reconstruction of the TM defect. Although temporalis fascia and autologous cartilage grafts can both be used effectively for the purpose, cartilage is preferred over temporalis graft, especially for large/subtotal perforations where a poor success rate is often anticipated. Like the temporalis fascia graft, autologous cartilage can be abundantly harvested from the concha or tragus without causing cosmetic deformity to the ear. A review of the literature reveals a number of studies previously conducted demonstrating the effectiveness of cartilage graft over the temporalis fascia for type-I tympanoplasty (1,2). In the present study, we have obtained encouraging postoperative results of cartilage palisade grafting over the temporalis fascia in type-I tympanoplasty. The graft uptake rate in patients with cartilage palisades was 96.7%, in contrast with the fascia group which had an uptake rate of 80% at the end of Month 24 (P=0.982). Similarly, the mean closure of the ABG in the fascia group was 15 dB, in contrast with the cartilage tympanoplasty group in which it was found to be 11 dB (P=0.512). In total, 90% of patients in the fascia group and 88% of patients in the cartilage palisade group showed significant improvement in hearing (ABG ≥10 dB), which is consistent with a previous study by Kalcioglu et al. (4). According to Gerber et al. (5), and also in the present study, there was significant improvement in the SRT in both groups of patients. The mean gain in SRT was 10 dB and 9 dB in the temporalis fascia group and cartilage palisade group, respectively. In total, 75% of patients in the temporalis fascia group and 60% of the cartilage palisade group had a significant improvement in SRT (≥10 dB), although the difference was not significant (P=0.473) (Fig.4). 

**Fig 5:**
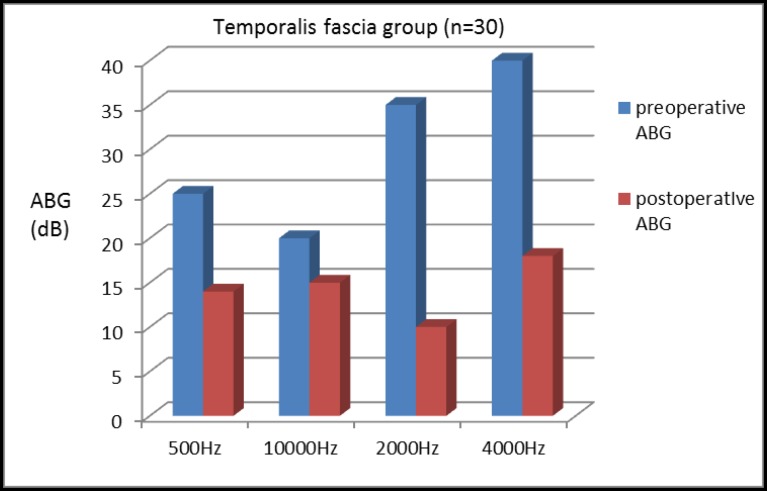
Postoperative closure of ABG in patients with temporalis fascia grafting

**Fig 6:**
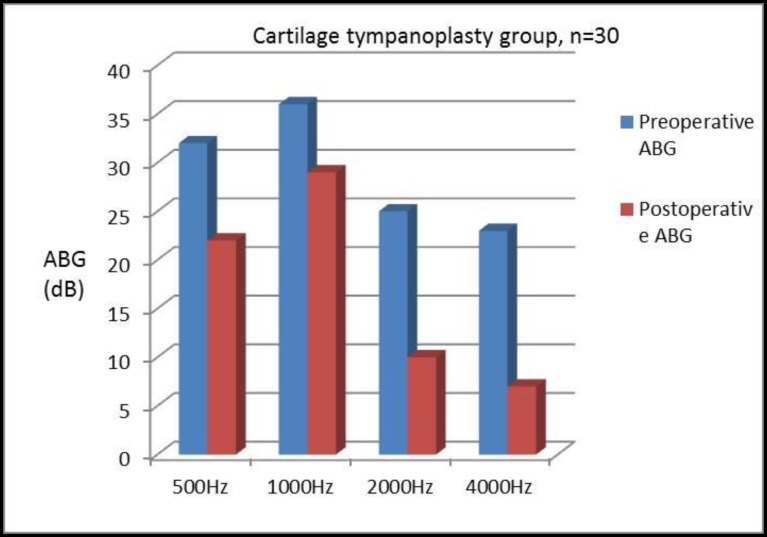
Postoperative closure of ABG in patients with cartilage palisade grafting

**Fig 7:**
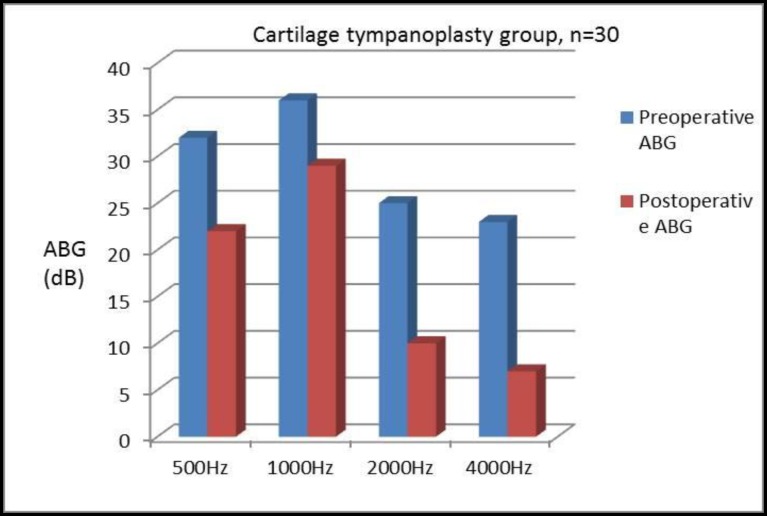
Postoperative closure of ABG compared between temporalis fascia and cartilage grafting

When considered at individual frequencies, it was found that patients in both groups had ABG closure at each frequency level, and was maximum toward the higher frequency in both the groups; although no significant difference was noticed between them (Fig.5-7).

These encouraging anatomical and functional results may be due to the nature of the cartilage palisades. As cartilage is nourished by simple diffusion from the surrounding tissue, it can survive in a relatively vascularized state and thus cartilage palisades may be a valid alternative to the conventional temporalis fascia graft for the reconstruction of large/subtotal perforation (3). Although it might be expected that full thickness cartilage would hamper the conduction of sound due to mass effect of the cartilage palisades, in our study the outcomes of the hearing assessments were actually satisfactory. Eighty-eight percent of patients presented with significant improvement in hearing (˃10 dB) in the postoperative period, which is consistent with a study conducted by Dornhoffer comparing the cartilage island and cartilage palisade technique for type-I and type-II tympanoplasties (6). Dornhoffer demonstrated that the anatomical and functional outcomes were similar in both the groups; i.e. ABG <10 dB was found in 70% of cases using the cartilage island technique and in 73% of cartilage palisades cases. Vashishth et al. also demonstrated the effectiveness of cartilage palisade over the temporalis fascia graft for the repair of near total/total perforations in revision and pediatric cases, and obtained better anatomical and functional results compared with patients in whom temporalis fascia was used (7). Similarly, Güneri et al., Ozbek et al. and Wielgosz et al. have also claimed a better graft uptake rate and hearing outcome in cartilage tympanoplasty compared with temporalis fascia in type-I tympanoplasty (8-10). Although construction of the cartilage assembly is somewhat difficult and time consuming, it yields predictable results both in terms of anatomical and functional considerations. There was no definite association between the tragal and conchal cartilage for type-I tympanoplasty, although it is believed that conchal cartilage is superior because of its concavity, similar to the normal TM. Further, there was no association between the surgical approaches (postaural/ endaural) toward the final outcomes of type-I tympanoplasty, although better patient compliance was achieved in the end using the aural approach.

## Conclusion

Repair of large or subtotal perforation is always a challenge to the otologist. Although temporalis fascia and tragal cartilage palisades can both effectively be used for the above purpose, cartilage graft is thought to be the superior autograft compared with temporalis fascia, not only because of better graft integration rate but also due to the comparable hearing outcomes. Although cartilage grafting is somewhat difficult and time consuming, it can be considered as the first-line treatment in COM with large/subtotal perforations, with satisfactory outcomes.

## References

[1] Iacovou E, Vlastarakos PV, Panagiotakopoulou A, Chrysostomou M, Kandiloros D, Adamopoulos G (2012). Effect of type I tympanoplasty on the resonant frequency of the middle ear: comparison between chondrotympanoplasty and fascia grafting. Otolaryngol Head Neck Surg.

[2] Kazikdas KC, Onal K, Boyraz I, Karabulut E (2007). Palisadecartilagetympanoplastyfor management of subtotal perforations: acomparisonwith thetemporalisfasciatechnique. Eur Arch Otorhino- laryngol.

[3] Sschuknecht H, Shi S (1985). Surgical pathology of middle ear implants. Laryngoscope.

[4] Kalcioglu MT, Tan M, Croo A (2013). Comparisonbetweencartilageandfasciagrafts intype1tympanoplasty. B-ENT.

[5] Gerber MJ, Mason JC, Lambert PR (2000). Hearing Results After Primary Cartilage Tympanopalsty. Laryngoscope.

[6] Dornhoffer JL (1997). Hearing results with cartilage tympanoplasty. Laryngoscope.

[7] Vashishth A, Mathur NN, Choudhary SR, Bhardwaj A (2014). Clinical advantages of cartilage palisades over temporalis fascia in type I tympano- plasty. Auris Nasus Larynx.

[8] Güneri EA, Ikiz AO, Erdağ TK, Sütay S (2009). Cartilage tympanoplasty: indications, techniques, and results. J Otolaryngol Head Neck Surg.

[9] Ozbek C, Ciftçi O, Tuna EE, Yazkan O, Ozdem C (2008). A comparison of cartilage palisades and fascia in type 1 tympanoplasty in children: anatomic and functional results. Otol Neurotol.

[10] Wielgosz R, Mroczkowski E (2006). Assessment of the hearing results in tympanoplasties with the use of palisade-technique. Otolaryngol Pol.

